# Editorial: Mitochondrial dysfunction and genetic variations in neuro-ophthalmology diseases

**DOI:** 10.3389/fopht.2024.1483607

**Published:** 2024-10-30

**Authors:** Neeru A. Vallabh, Ian Trounce

**Affiliations:** ^1^ Department of Eye and Vision Sciences, Institute of Life Course and Medical Sciences, University of Liverpool, Liverpool, United Kingdom; ^2^ St Paul's Eye Unit, Liverpool University Hospital Foundation Trust, Liverpool, United Kingdom; ^3^ Centre for Eye Research Australia, Ophthalmology, Department of Surgery, University of Melbourne, Liverpool, United Kingdom

**Keywords:** mitochondria, mtDNA, OXPHOS, ophthalmic disease, glaucoma, retina

This Research Topic brings together five articles investigating mitochondrial genetic and functional contributions to neuro-ophthalmological disorders. Retinal neurons, including retinal ganglion cells (RGCs), photoreceptors, and the retinal pigment epithelium, are highly energetic cells dependent on adequate ATP supply from mitochondria. The mitochondrial energetic machinery of oxidative phosphorylation (OXPHOS) has polypeptide components encoded in both nuclear genes and the mitochondrial DNA (mtDNA). Leber’s hereditary optic neuropathy (LHON) and autosomal dominant optic atrophy (ADOA) are inherited mitochondrial optic neuropathies that are characterized by the selective neurodegeneration of RGCs. In addition to these classic mitochondrial optic neuropathies, studies have indicated mitochondrial involvement in Age- Related Macular Degeneration, inherited retinal diseases, and glaucoma (1–4).

The mitochondrial genome can replicate independently of nuclear DNA, and there are usually 100–10,000 copies of mtDNA present in each cell in humans (5). The mitochondrial genome is organized as a circular, double-stranded DNA molecule and codes for only 37 genes across ~16,600 base pairs. Homoplasmy describes the state when all copies of mtDNA are mutant and heteroplasmy is the state when only a proportion of the mtDNA is mutant (5). Therefore, the coexistence of multiple mtDNA species (wild- type mtDNA and mutated mtDNA variants) in a single cell or among cells within an individual makes mitochondrial diseases more complex and heterogeneous.

The status of research into the pathogenesis and therapy of LHON is reviewed by (Esmaeil et al.). They provide an overview of the prevalence of LHON, the established mtDNA mutations in OXPHOS complex I genes, and ongoing controversies around bioenergetic insufficiency versus oxidative stress as primary pathogenic drivers. In addition, they consider the clinical course of the condition, along with other extraocular features and potential links to neurological disorders. They conclude with an update on ongoing gene therapy trials in LHON that show promise.

Some phenotypic similarities between classic mitochondrial optic neuropathies and glaucoma have prompted research into potential mitochondrial contributions to glaucoma, the most common optic neuropathy. Studies have found evidence of mitochondrial functional and mtDNA genetic contributions to glaucoma (2, 6). The report from Vallbona-Garcia et al. adds to this by showing that mtDNA D-loop homoplasmic variants appear to be increased in high- tension glaucoma subjects with a higher percentage of those being in the 7S DNA within the HV1 region. As the mtDNA D-loop is noncoding but contains regions crucial to the regulation of mtDNA replication and transcription, they speculate that some of these variants may lead to mtDNA copy number depletion, which they have previously identified in this sub-group of glaucoma (7). An improved understanding of changes in mitochondrial replication may be vital for determining the role of treatments that target mitochondrial biogenesis as potential future strategies for neuro-ophthalmological disorders.

Reflecting disparities in access to health care and participation in clinical studies, individuals of African ancestry have been underrepresented in retinal research as participants. Patients of African origin are disproportionately affected by primary open angle glaucoma (POAG) at all ages; the prevalence of POAG in African- Americans is fivefold higher than in Caucasians. The biological mechanisms by which this group are disproportionately affected is poorly understood. Kuang et al. bring the current evidence of mtDNA analyses of glaucoma subjects of African ancestry into focus. African ancestry presents the largest diversity of mtDNA variation in humans, due to this continent being the source of human dispersal in prehistory. As such, this diversity requires large and carefully matched cohorts for genetic analysis. Kuang et al. summarize the associations found to date, with non-L3 mtDNA L haplogroups showing higher prevalence in glaucoma subjects. The establishment of the Primary Open Angle African American Glaucoma Genetics study (POAAGG) in Philadelphia has already uncovered new nuclear genetic loci associated with glaucoma risk in this population (8); we eagerly await mtDNA analysis in this largest African ancestry population cohort to date.

Some specialized retinal imaging technologies may be well suited to detecting mitochondrial dysfunction *in vivo* (9). Among these, flavoprotein fluorescence (FPF) holds promise. Both NAD/H and FAD/H are key intermediaries of OXPHOS and, due to their intrinsic fluorescence, their measurement can provide information on mitochondrial metabolic alterations and oxidative stress. Ahsanuddin et al. show that current- generation FPF retinal cameras can robustly discriminate diseased eyes with various retinal diseases associated with oxidative stress, including exudative macular degeneration, diabetic retinopathy, and retinal vein occlusion. Imaging mitochondrial dysfunction *in vivo* using flavoprotein fluorescence may enable real-time, non-invasive assessment of cellular metabolic health, potentially leading to earlier detection and determining those at greater risk of progression. This technology holds great promise in parsing mitochondrial endophenotypes within diverse disease groupings such as glaucoma (10).

Rombaut et al. go beyond the retinal ganglion cell to explore evidence for wider metabolic alterations in retinal glia cells in glaucoma. Retinal glial cells support and protect retinal ganglion cells by maintaining the structural integrity of the retina, regulating neurotransmitter levels, and managing the retinal environment to ensure proper visual function. They summarize evidence for alterations in glaucoma of the neuro-glia metabolic coupling, including in retinal Muller cells, microglia, and astrocytes. The authors present an authoritative perspective on NAD balance, the lactate shuttle, glutamate handling, and lipid- shuttling roles of glia in support of retinal neurons and how impacts on these cells may in turn influence neuronal health and survival where mitochondria sit as a central hub. Therefore, retinal glial cells may also provide shared neuroprotective targets for glaucoma treatment.

Mitochondrial studies in ophthalmic diseases continue to gain momentum. The relative ease of access to the eye for ocular samples, imaging, and gene therapy puts retinal diseases at the forefront of mitochondrial therapeutic development in neuroscience. The overlap of neurodegenerative brain and eye diseases with mitochondrial dysfunction promises to be a productive research focus. Mitochondria are a central metabolic hub, and the added twist of having their own maternally inherited genome brings complexity to genetic analyses that are only recently being appreciated. The integration of mitochondrial genetics and function in neuro-ophthalmological research holds promise for advancing our understanding and providing translational personalized treatments of these disorders.
